# Statins Activate Human PPAR*α* Promoter and Increase PPAR*α* mRNA Expression and Activation in HepG2 Cells

**DOI:** 10.1155/2008/316306

**Published:** 2008-12-24

**Authors:** Makoto Seo, Ikuo Inoue, Masaaki Ikeda, Takanari Nakano, Seiichiro Takahashi, Shigehiro Katayama, Tsugikazu Komoda

**Affiliations:** ^1^Department of Biochemistry, Faculty of Medicine, Saitama Medical University, Saitama 350-0495, Japan; ^2^Department of Endocrinology and Diabetes, Faculty of Medicine, Saitama Medical University, Saitama 350-0495, Japan; ^3^Department of Physiology, Faculty of Medicine, Saitama Medical University, Saitama 350-0495, Japan; ^4^Molecular Clock Project, Research Center for Genomic Medicine, Saitama Medical University, Saitama 350-0495, Japan

## Abstract

Statins increase peroxisome proliferator-activated receptor *α* (PPAR*α*) mRNA expression, but the mechanism of this increased PPAR*α* production remains elusive. To examine the regulation of PPAR*α* production, we examined the effect of 7 statins (atorvastatin, cerivastatin, fluvastatin, pitavastatin, pravastatin, rosuvastatin, and simvastatin) on human PPAR*α* promoter activity, mRNA expression, nuclear protein levels, and transcriptional activity. The main results are as follows. (1) Majority of statins enhanced PPAR*α* promoter activity in a dose-dependent manner in HepG2 cells transfected with the human PPAR*α* promoter. This enhancement may be mediated by statin-induced HNF-4*α*. (2) PPAR*α* mRNA expression was increased by statin treatment. (3) The PPAR*α* levels in nuclear fractions were increased by statin treatment. (4) Simvastatin, pravastatin, and cerivastatin markedly enhanced transcriptional activity in 293T cells cotransfected with acyl-coenzyme A oxidase promoter and PPAR*α*/RXR*α* expression vectors. In summary, these data demonstrate that PPAR*α* production and activation are upregulated through the PPAR*α* promoter activity by statin treatment.

## 1. INTRODUCTION

Statins, 3-hydroxy-3-methylglutaryl coenzyme A (HMG-CoA) reductase inhibitors, are the most widely used drugs to lower low-density lipoprotein (LDL) cholesterol. These mechanisms have been reported that treatment with statins results in lowering intracellular cholesterol concentration, and then increasing a proteolytic activation of sterol responsive element-binding proteins (SREBPs) [1]. These transcription factors increase the cholesterol homeostasis controlling genes, such as LDL receptor, lipoprotein lipase, and cholesterol 7*α*-hydroxylase [2, 3].

Currently, statins are the first choice of therapeutic agent for the treatment of hyperlipidemia. Several mega trials and large cohort studies using statins have shown that statins prevent coronary heart disease and decrease the incidence of cardiovascular events [4–6]. The reasons why cardiovascular events were decreased with statins are reported to be due to many pleiotropic effects, for example, inhibition of the proliferation and migration of endothelial cells, smooth muscle cells, and macrophages [7, 8]. Moreover, statins up-regulate the expression of endothelial nitric oxide synthesis [9] and suppress oxidative stress, as seen in the reduced formation of reactive oxygen species and p22^phox^ expression [10, 11].

The peroxisome proliferator-activated receptors (PPARs) belong to the nuclear receptor superfamily and play an important role in the regulation of lipid and glucose metabolism and adipocyte differentiation [12, 13]. PPAR*α* is expressed in the liver, kidney, heart, and muscle where it regulates energy homeostasis. PPAR*α* forms a heterodimer with retinoid X receptor *α* (RXR*α*), which enhances its binding to peroxisome proliferator response elements (PPREs) and activates target genes. PPAR*α* activates the uptake and catabolism of fatty acids that result in a decrease of triglyceride (TG), stimulate gluconeogenesis, and enhance high-density lipoprotein synthesis [14, 15]. Fibrates, which are a ligand for PPAR*α*, have been reported to lower the serum TG levels [16]. Some statins were also reported to decrease the serum TG levels to same extent [17–19]. Although it is reported that several statins increase PPAR*α* [20, 21], it is not clear how statins regulate nuclear transcription, and PPAR*α* mRNA expression and activity. Previously, simvastatin activated mouse PPAR*α* promoter and induced the transcription of PPAR*α* gene [22], but there is no report that statins activate the human PPAR*α* promoter and transcription of this gene.

In the present study, we investigated the effect of 7 statins (atorvastatin, cerivastatin, fluvastatin, pitavastatin, pravastatin, rosuvastatin, simvastatin) on the regulation of PPAR*α* mRNA expression and PPAR*α* protein levels in nuclear fraction of the human hepatoblastoma cell line (HepG2 cells). We also investigated the effect of statin treatment on the promoter activity of the human PPAR*α* gene. In addition, we investigated whether statin treatment could induce transcriptional activity of PPAR*α*.

## 2. MATERIALS AND METHODS

### 2.1. Reagents and cell culture

Seven statins were kindly provided as follows; atorvastain (Warner-Lambert Co., Ltd.), cerivastatin (Bayel Co., Ltd.), fluvastatin (Novartis Co., Ltd.), pitavastatin (Kowa Co., Ltd.), pravastatin (Sankyo Co., Ltd.), and rosuvastatin (AstraZeneca Ltd.). Simvastatin was purchased from Wako Pure Chemical Industries, Ltd. (Tokyo, Japan). Fenofibric acid (FA) was kindly provided by Kaken Pharmaceutical Co., Ltd. Atorvastatin, cerivastatin, fluvastatin, pitavastatin, rosuvastatin, and FA were dissolved in dimethyl sulfoxide (DMSO); simvastatin was dissolved in ethanol, and pravastatin was dissolved in distilled water. In all assays, the final concentrations of DMSO and ethanol were less than 0.5%. HepG2 cells was purchased from JCRB (cell number: JCRB1054) and human kidney 293T cells (293T cells) from Dainippon Pharmaceutical Co., Ltd. They were cultured in Dulbecco's modified Eagle's medium (DMEM) (Invitrogen) containing 10% heat-inactivated fetal bovine serum (FBS) (JRH Biosciences) and PNS antibiotic mixture (Invitrogen) at 37°C in 5% CO_2_.

### 2.2. Cloning of the PPAR*α* promoter and plasmid constructions

To generate human PPAR*α* promoter-reporter plasmid, we referred to the genomic sequence that has been reported previously [23]. Human PPAR*α* promoter containing −1553 bp to +88  bp was obtained by polymerase chain reaction (PCR) with human genomic DNA (Clontech) using a forward primer 5′-CATAAGCTTACCCCACGAGATATGCAGGAT-3′ (including a *Hind* III site, underlined) and a reverse primer 5′-CGTAAGCTTCGCAAGAGTCCTCGGTGTGT-3′ (including a *Hind* III site, underlined). This promoter was cloned into the *Hind* III site of a pGL3-Basic vector (Promega). Plasmid DNA used for transfection was prepared using the Wizard *Plus* Minipreps DNA Purification System (Promega). Nucleotide sequences of this plasmid were confirmed by sequencing using ABI PRISM 310 Genetic Analyzer (Applied Biosystems).

### 2.3. Luciferase assay of PPAR*α* promoter activity

HepG2 cells were transfected using Lipofectamine 2000 (Invitrogen) according to the manufacturer's protocols. The cells (1 × 10^5^ cells/well) were seeded in 24-well plates (Falcon) and incubated for 18 hours before transfection. The cells were transfected with the use of Lipofectamine 2000 with 1 *μ*g of human PPAR*α* promoter-reporter plasmid and 0.1 *μ*g of pRL-TK (Promega), a *renilla* luciferase reporter vector as internal control for transfection efficiency. After 3 hours, the transfection medium was replaced by 10% FBS-DMEM plus the various amounts of statin (0, 1, 10, 25, and 50 *μ*M) or vehicle (DMSO, ethanol, or distilled water) and the cells were incubated for 24 hours. Luciferase activities were quantified using a Dual-Luciferase Reporter Assay System (Promega) according to the manufacturer's protocols.

### 2.4. Real-time reverse transcription (RT)-PCR analysis

HepG2 cells (2 × 10^5^ cells/dish) were incubated with various amounts of statin (0, 5, 10, and 20 *μ*M, pravastatin was 50, 100, and 250 *μ*M) at 37°C for 24 hours. After treatment with statins, cells were homogenized in 1 mL of ISOGEN (Nippongene), and then total RNA was extracted with chloroform and precipitated with ethanol. First-strand cDNA was generated from total RNA with random hexamers and MuLV transcriptase (Applied Biosystems) according to the manufacturer's protocols. PCR reactions were performed with TaqMan Universal PCR Master Mix and TaqMan Gene Expression Assays (Applied Biosystems). Identification numbers of the assay mixture of target gene-specific primers and probes were as follows: human PPAR*α*, Hs00231882_m1; 18S ribosomal RNA (house-keeping gene), Hs99999901_s1. Real-time PCR reactions were performed with thermal cycling conditions of 2 minutes at 50°C, 10 minutes at 95°C, and 40 cycles of 15 seconds at 95°C and 1 minute at 60°C using ABI PRISM 7900HT Sequence Detection System (Applied Biosystems). PPAR*α* mRNA levels were normalized to 18S ribosomal RNA levels, and are presented as fold difference of statin-treated cells compared with untreated cells.

### 2.5. Western blot analysis

HepG2 cells (2 × 10^5^ cells/dish) were seeded in 60 mm dishes (Falcon) and incubated for 18 hours. Then, the cells were incubated with 10 and 25 *μ*M statin at 37°C for 24 hours. After treatment with statins, cells were washed with ice-cold phosphate buffered saline and collected. After centrifugation (15,000×g), the cytoplasmic and nuclear proteins of the cells were extracted with NE-PER Nuclear and Cytoplasmic Extraction Reagents (PIERCE) according to the manufacturer's protocols and the proteins concentration was determined with a BCA Protein Assay kit (PIERCE). Aliquots (15 *μ*g) of cytoplasmic or nuclear proteins were electrophoresed on 9% sodium dodecyl sulfate-polyacrylamide gels and transferred to polyvinylidene difluoride membranes (Millipore). The membranes were blocked with BlockingOne (Nacalai Tesque, Inc.), and incubated overnight with goat anti-PPAR*α* IgG antibody (sc-1985, Santa Cruz) (diluted 1:1000 with BlockingOne) or mouse anti-hepatocyte nuclear factor-4*α* (HNF-4*α*) IgG antibody (Clone no.: H1415, Perseus Proteomics Co., Ltd.). After washing four times with Tris-buffered saline-containing 0.5% Tween 20, signals from Western blots were obtained using horseradish peroxidase-conjugated secondary anti-goat antibody (diluted 1:2000 with BlockingOne) and visualized with the ECL detection system (Amersham Biosciences). The PPAR*α* protein levels were quantified with an imaging analyzer (Densitograph, ATTO). The data are expressed as % of control.

### 2.6. Luciferase assay of PPRE activity

Constructions of pCI-PPAR*α* and pCI-RXR*α* expression plasmids were described previously [24]. Briefly, the full-length human PPAR*α* (GenBank accession no. L_02932) and human RXR*α* (GenBank accession no. X_52773) were prepared by PCR. The specific DNA fragmant of human PPAR*α* was cloned into the *SalI-NotI* sites of the pCI-neo mammalian expression vector (Promega). The human RXR*α* was also cloned into the *XhoI-NotI* sites of the pCI-neo. The human acyl-coenzyme A oxidase (AOX) promoter (GenBank accession no. NT_010641) construct containing the PPREs was previously cloned into the *KpnI-NcoI* sites of pGL3-Basic vector [25].

To measure the transcriptional activation of PPRE, 293T cells (0.5 × 10^5^ cells/well) were seeded in collagen type I-coated 24-well plate (Iwaki) and incubated for 18 hours before transfection. The cells were transfected using Lipofectamine 2000 with 0.5 *μ*g of human AOX promoter-reporter plasmid, 0.1 *μ*g of pRL-TK as internal control for transfection efficiency and either 0.25 *μ*g of pCI-PPAR*α* and pCI-RXR*α* expression vectors or 0.5 *μ*g of pCI-neo vector. After 3 hours, the transfection medium was replaced by 10% FBS-DMEM plus the various amounts of statin (0, 1, 10, 25, and 50 *μ*M), fenofibric acid (0, 1, 10, 50, 100 *μ*M), or vehicle (DMSO, ethanol, or distilled water) and the cells were incubated for 24 hours. Luciferase activities were quantified using a Dual-Luciferase Reporter Assay System (Promega) according to the manufacturer's protocols.

### 2.7. Statistical analysis

All data are presented as the means ± SEM. Statistical analysis was performed using ANOVA followed by the Dunnett test or Scheffe test (StatView software). Statistical significance was considered as *P* < .05.

## 3. RESULTS

### 3.1. Statins increased PPAR*α* mRNA expression in HepG2 cells

**Figure 1:**
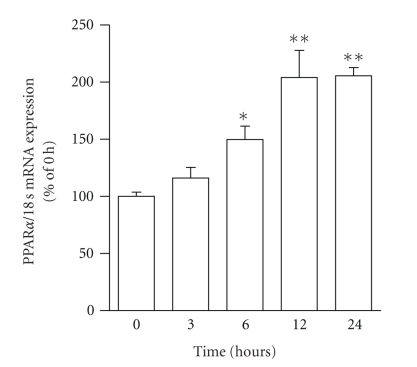
Time-course of PPAR*α* mRNA expression in HepG2 cells after treatment with 10 *μ*M simvastatin. The data are expressed as % of controls at 0 hour. Values are presented as the mean ± SEM of three separate experiments, significantly different from control at *P < .05, **P < .01.

We first examined the effect of simvastatin on the PPAR*α* mRNA expression in HepG2 cells. The time-course study for the PPAR*α* mRNA expression in HepG2 cells treated with 10 *μ*M simvastatin is shown in Figure 1. Simvastatin significantly increased PPAR*α* mRNA expression by 2.0-fold (versus the control) at 12 and 24 hours.

**Figure 2:**
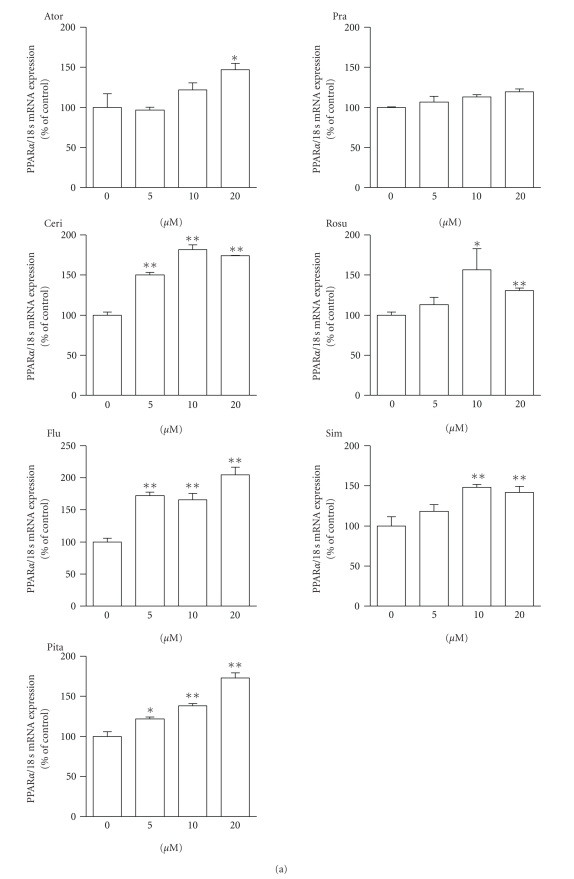

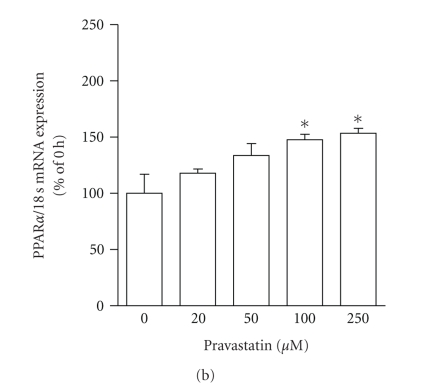
PPAR*α* mRNA expression in HepG2 cells after treatment with atorvastatin (Ator), cerivastatin (Ceri), fluvastatin (Flu), pitavastatin (Pita), pravastatin (Pra), rosuvastatin (Rosu), and simvastatin (Sim) for 24 hours. (a) Each statin was used at concentrations of 5, 10, and 20 *μ*M. Nontreated cells (statin concentration 0 *μ*M) were the control. (b) Pravastatin was used at higher concentrations of 20, 50, 100, and 250 *μ*M. Nontreated cells (pravastatin concentration 0 *μ*M) were the control. The data are expressed as % of controls. Values are presented as the mean ± SEM of three separate experiments, significantly different from control at *P < .05, **P < .01.

We next examined the effect of atorvastatin, cerivastatin, fluvastatin, pitavastatin, pravastatin, rosuvastatin, and simvastatin for 24 hours on PPAR*α* mRNA expression in HepG2 cells. PPAR*α* mRNA expression following treatment of HepG2 cells with various amounts of statin is shown in Figure 2. In Figure 2(a), atorvastatin (20 *μ*M), cerivastatin (5, 10, and 20 *μ*M), fluvastatin (5, 10, and 20 *μ*M), pitavastatin (20 *μ*M), rosuvastatin (10 *μ*M), and simvastatin (10 *μ*M) significantly increased PPAR*α* mRNA expression by more than 1.5-fold (versus the control). Pravastatin did not increase PPAR*α* mRNA expression at these concentrations, but the higher concentrations of pravastatin-treatment (100 and 250 *μ*M) significantly increased PPAR*α* mRNA expression (Figure 2(b)).

### 3.2. Statins increased human PPAR*α* promoter activity

**Figure 3:**
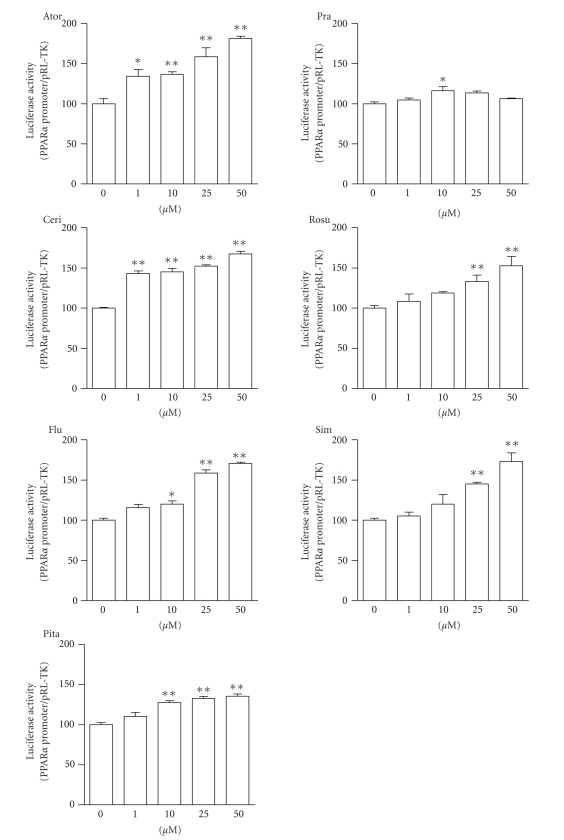
PPAR*α* promoter activity in HepG2 cells transfected with human PPAR*α* promoter-reporter plasmid after treatment with atorvastatin (Ator), cerivastatin (Ceri), fluvastatin (Flu), pitavastatin (Pita), pravastatin (Pra), rosuvastatin (Rosu), and simvastatin (Sim) for 24 hours. Each statin was used at doses of 1, 10, 25, and 50 *μ*M. Nontreated cells (statin concentration 0 *μ*M) were the control. The data are expressed as % of controls. Values are presented as the mean ± SEM of three separate experiments, significantly different from control at *P < .05, **P < .01.

To investigate the mechanism by which statins increase PPAR*α* mRNA expression, we cloned the human PPAR*α* promoter region (−1553 to +88 bp) and examined promoter activity in HepG2 cells transfected with the human PPAR*α* promoter-reporter plasmid. Figure 3 shows the PPAR*α* promoter activity following treatment of HepG2 cells with various amounts of statin for 24 hours. Except for pravastatin, 6 statins significantly increased PPAR*α* promoter activity in a dose-dependent manner. Atorvastatin, cerivastatin, fluvastatin, rosuvastatin, and simvastatin increased PPAR*α* promoter activity by more than 1.5-fold (versus the control). However, pravastatin only slightly increased PPAR*α* promoter activity that was significant only at 10 *μ*M.

### 3.3. Statins increased PPAR*α* levels in nuclear fraction

**Figure 4:**
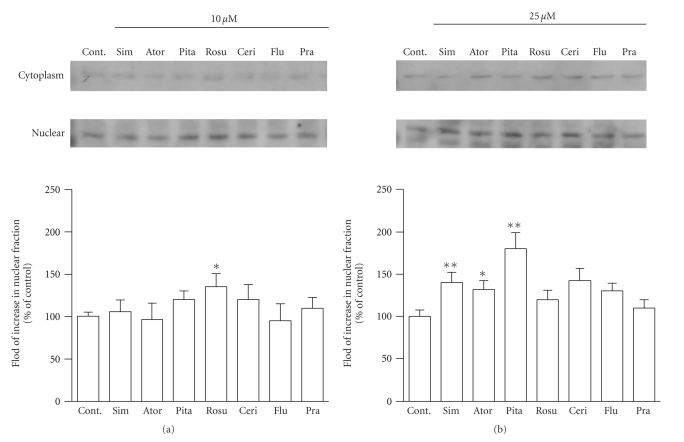
The Western blots represent PPAR*α* protein levels in nuclear fractions of HepG2 cells after treatment with 10 *μ*M (a) and 25 *μ*M (b) of statin for 24 hours (Cont., control; Ator, atorvastatin; Ceri, cerivastatin; Flu, fluvastatin; Pita, pitavastatin; Pra, pravastatin; Rosu, rosuvastatin; Sim, simvastatin). The PPAR*α* protein levels were quantified with an imaging analyzer. The data are expressed as % of control. Values are presented as the mean ± SEM of three separate experiments, significantly different from control at *P < .05, **P < .01.

We next examined the increasing effect of statins on PPAR*α* protein levels in nuclear fraction of HepG2 cells. Results are shown in Figure 4. In a nuclear fraction of HepG2 cells treated with 10 *μ*M statin, PPAR*α* protein levels were significantly increased by treatment with rosuvastatin (Figure 4(a)). Moreover, PPAR*α* protein levels were significantly increased by treatment with 25 *μ*M of pitavastatin, simvastatin, and atorvastatin, and the other statins slightly increased PPAR*α* protein levels (Figure 4(b)). However, in a cytoplasmic fraction, PPAR*α* protein levels were not changed by the treatment with 10 and 25 *μ*M statins.

### 3.4. Statins increased PPAR*α* activity

**Figure 5:**
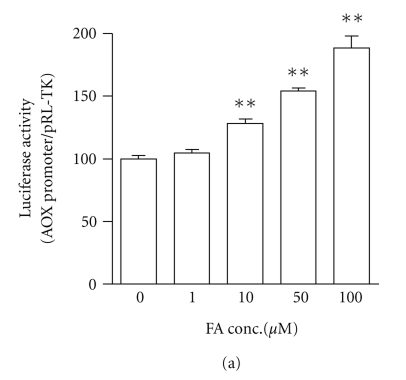

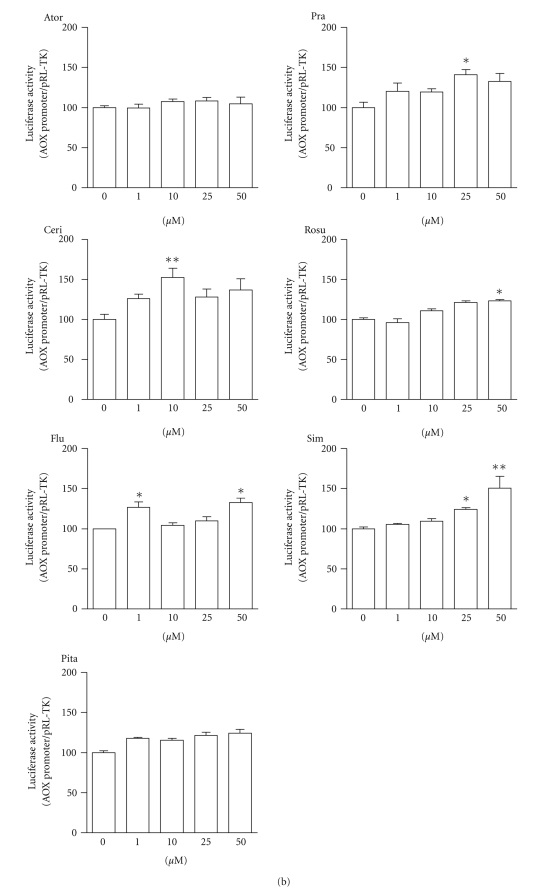
The transcriptional activity of PPAR*α* in HepG2 cells transfected with human acyl-CoA oxidase (AOX) promoter-reporter plasmid after treatment with fenofibric acid (FA) and statins for 24 hours. (a) FA was used at doses of 1, 10, 50, and 100 *μ*M. Nontreated cells (FA concentration 0 *μ*M) were the control. (b) Atorvastatin (Ator), cerivastatin (Ceri), fluvastatin (Flu), pitavastatin (Pita), pravastatin (Pra), rosuvastatin (Rosu), and simvastatin (Sim) for 24 hours. Each statin was used at doses of 1, 10, 25, and 50 *μ*M. Nontreated cells (statin concentration 0 *μ*M) were the control. The data are expressed as % of control. Values are presented as the mean ± SEM of three separate experiments, significantly different from control at *P < .05, **P < .01.

We next examined the effect of statins on the transcriptional activity of PPAR*α* in 293T cells transfected with human AOX promoter-reporter plasmid containing PPREs region, human PPAR*α*, and RXR*α* expression plasmids. In Figure 5(a), fenofibric acid that was used as a positive control increased PPAR*α* activity in a dose-dependent manner. In Figure 5(b), the treatment with cerivastatin (10 *μ*M) and simvastatin (50 *μ*M) significantly increased transcriptional activity of PPAR*α* by more than 1.5-fold (versus the control). Fluvastatin, pitavastatin, pravastatin, and rosuvastatin tended to increase transcriptional activity of PPAR*α* by 1.2- to 1.4-fold (versus the control). However, atorvastatin did not increase the transcriptional activity of PPAR*α*.

### 3.5. Statins increased HNF-4*α* levels in nuclear fraction

**Figure 6:**
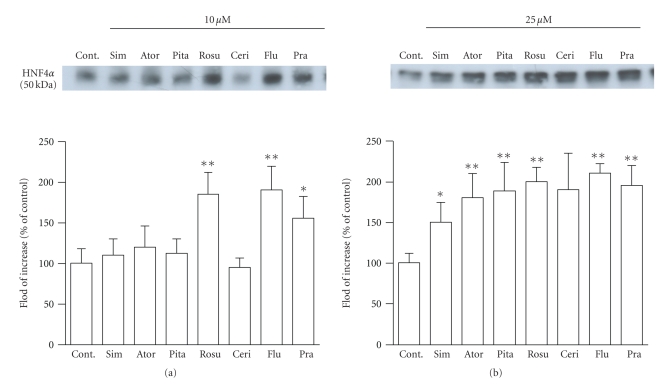
The Western blots represent HNF-4*α* levels in nuclear fractions of HepG2 cells after treatment with atorvastatin (Ator), cerivastatin (Ceri), fluvastatin (Flu), pitavastatin (Pita), pravastatin (Pra), rosuvastatin (Rosu), and simvastatin (Sim) for 24 hours. Each statin was used at doses of 10 (a) or 25 (b) *μ*M. Nontreated cells (statin concentration 0 *μ*M) were the control. HNF-4*α* protein levels were quantified with an imaging analyzer. The data are expressed as % of control. Values are presented as the mean ± SEM of three separate experiments, significantly different from control at *P < .05, **P < .01.

Next, to elucidate the downstream effects of statins on transcriptional activation by PPAR*α*, we detected HNF-4*α* levels in nuclear fraction of HepG2 cells treated with statins by the use of Western blot analysis. Results are shown in Figure 6. At 10 *μ*M statin treatment, fluvastatin, pravastatin, and rosuvastatin significantly increased HNF-4*α* levels in nuclear fraction (Figure 6(a)). Moreover, at 25 *μ*M statin treatment, except for cerivastatin, 6 statins significantly increased HNF-4*α* levels in nuclear fraction (Figure 6(b)).

## 4. DISCUSSION

The main findings of the present study were (1) most statins increased PPAR*α* mRNA expression, which might be caused via PPAR*α* promoter activation, (2) atorvastatin, pitavastatin, and simvastatin significantly increased PPAR*α* protein levels in nuclear fraction, (3) some, not but all, statins interacted with AOX promoter containing PPRE and increased PPAR*α* activity, and (4) the PPAR*α* promoter activity could be regulated by the increase of statin-induced HNF-4*α*.

Statin therapy has been reported to reduce the incidence of cardiovascular disease risk in patients with the metabolic syndrome and hyperlipidemia [26], and these benefits have been regarded to mainly derive from their lipid-lowering effect. However, recent studies have suggested that there are additional, beneficial anti-inflammatory effects of stains, which are independent of their cholesterol-lowering effect [27, 28]. There are many reports that the anti-inflammatory effects of statins are induced via PPARs signaling-pathway [11, 29].

Our present results show that most statins increased PPAR*α* mRNA expression in HepG2 cells after 24 hours treatment, especially atorvastatin, cerivastatin, fluvastatin, pitavastatin, rosuvastatin, and simvastatin (more than 1.5-fold versus control). Statins are classified into hydrophilic compounds and lipophilic compounds. In this study, the majority of the statins are lipophilic compounds, but pravastatin and rosuvastatin are hydrophilic compounds. Our results of PPAR*α* mRNA expression in HepG2 cells treated with statins show that higher concentrations of pravastatin (100 and 250 *μ*M) significantly increased PPAR*α* mRNA expression. Therefore, in hydrophilic statin (pravastatin), the higher concentrations compared with other statins would be required for increase PPAR*α* mRNA expression.

There are many reports that statins increase PPAR*α* mRNA expression [11, 21]; however, there are no reports about the effect of statins on human PPAR*α* promoter activity. We, therefore, cloned the human PPAR*α* promoter region (−1553  bp to +88  bp) and measured the promoter activity in HepG2 cells treated with statins. Our present results show that 6 statins (except for pravastatin) significantly increased PPAR*α* promoter activity in a dose-dependent manner. Although the effect of statins on mouse PPAR*α* promoter activity has been reported previously [22], our present study is the first to report the effect of statins on human PPAR*α* promoter activity.

PPAR*α* promoter region includes many transcription factor binding domains, such as HNF-4*α*, PPRE, E-Box, early growth response factor (Egr-1), and transcription factor Sp1. HNF-4*α* is a nuclear receptor that plays a key role in liver-specific gene expression. Previously, it was reported that human PPAR*α* promoter region contains HNF-4*α* response element (−1, 492  bp to −1, 483  bp), and HNF-4*α* induces human PPAR*α* promoter activity [23]. Therefore, we detected HNF-4*α* levels in nuclear fraction of statin-treated HepG2 cells. In our present studies, all statins (25 *μ*M) significantly increased HNF-4*α* in nuclear fractions. This result shows that statins may regulate PPAR*α* gene transcription mediated by downstream transcriptional factors (e.g., HNF-4*α*). Further studies will be necessary to elucidate molecular mechanisms of statins to regulate the other transcriptional factors related to PPAR*α* gene transcription.

Previously, we reported that cerivastatin, fluvastatin, and simvastatin increased nuclear translocation of PPAR*α* protein [11]. Our present results show that the 7 statins utilized in the present studies increased nuclear translocation of PPAR*α* protein in HepG2 cells compared with nontreated control cells. We next examined the effect of statins on transcriptional analysis of human AOX promoter in 293T cells cotransfected with human PPAR*α* and RXR*α* expression vector. 293T cells were used for these studies expressed very low levels of endogenous PPAR*α* production when treated with statins (data not shown). Our present results show that simvastatin increased human AOX promoter-transcriptional activity via PPAR*α*/RXR*α* heterodimer. In fact, we identified the upregulation of human AOX mRNA on HepG2 cells and 293T cells treated with statins (data not shown). PPAR*α* is a ligand-activated transcription factor and is activated by fatty acid, arachidonic acid [30], and several fibric acids [31]. PPAR*α*-dependent transcriptional activation of many genes is well documented, and direct, ligand-enhanced interactions between PPAR*α* and the coactivators, p300/cAMP-response element-binding protein (CREB-) binding protein (p300/CBP), steroid receptor coactivator-1 (SRC-1), PPAR-binding protein (PBP), and PPAR*γ* coactivator-1 (PGC-1) are thought to play a role in PPAR*α* activation [32–34]. The recruitment of specific coactivators and the release of corepressors (e.g., nuclear receptor corepressor, NCoR) that associate with the liganded PPAR*α*/RXR*α* heterodimer allow further fine control of gene transcription. PPAR*α*/RXR*α* heterodimer can also bind to PPRE in the unliganded state [35]. The molecular structures of the PPAR*α*/RXR*α* heterodimeric complex and coactivators remain to be elucidated. Further studies will be necessary to be undertaken of the molecular mechanisms of statin regulation of the gene transcription by binding to PPREs in the promoter region of target genes.

In conclusion, statins activate PPAR*α* promoter and then up-regulate PPAR*α* mRNA expression in HepG2 cells. The effect on PPAR*α* transcription is likely regulated by various downstream transcriptional factors (e.g., HNF-4*α*). Statins increase PPAR*α* protein levels in nuclear fraction, and moreover, some statins, such as cerivastatin, fluvastatin, and simvastatin, significantly activate the transcription of the PPAR*α* target genes.

## References

[1] Brown MS, Goldstein JL (1999). A proteolytic pathway that controls the cholesterol content of membranes, cells, and blood. *Proceedings of the National Academy of Sciences of the United States of America*.

[2] Fan P, Zhang B, Kuroki S, Saku K (2004). Pitavastatin, a potent hydroxymethylglutaryl coenzyme a reductase inhibitor, increases cholesterol 7 *α*-hydroxylase gene expression in HepG2 cells. *Circulation Journal*.

[3] Saiki A, Murano T, Watanabe F, Oyama T, Miyashita Y, Shirai K (2005). Pitavastatin enhanced lipoprotein lipase expression in 3T3-L1 preadipocytes. *Journal of Atherosclerosis and Thrombosis*.

[4] Tonkin A, Aylward P, Colquhoun D (1995). Design features and baseline characteristics of the LIPID (long-term intervention with pravastatin in ischemic disease) study: a randomized trial in patients with previous acute myocardial infarction and/or unstable angina pectoris. *The American Journal of Cardiology*.

[5] Ballantyne CM, Olsson AG, Cook TJ, Mercuri MF, Pedersen TR, Kjekshus J (2001). Influence of low high-density lipoprotein cholesterol and elevated triglyceride on coronary heart disease events and response to simvastatin therapy in 4S. *Circulation*.

[6] Streja L, Packard CJ, Shepherd J, Cobbe S, Ford I (2002). Factors affecting low-density lipoprotein and high-density lipoprotein cholesterol response to *pravastatin* in the West of Scotland Coronary Prevention Study (WOSCOPS). *The American Journal of Cardiology*.

[7] Laufs U, Marra D, Node K, Liao JK (1999). 3-hydroxy-3-methylglutaryl-CoA reductase inhibitors attenuate vascular smooth muscle proliferation by preventing Rho GTPase-induced down-regulation of p27^*K**i**p*1^. *The Journal of Biological Chemistry*.

[8] Takemoto M, Kitahara M, Yokote K (2001). NK-104, a 3-hydroxy-3-methylglutaryl coenzyme A reductase inhibitor, reduces osteopontin expression by rat aortic smooth muscle cells. *British Journal of Pharmacology*.

[9] Ding Q, Hayashi T, Packiasamy AJ (2004). The effect of high glucose on NO and O2- through endothelial GTPCH1 and NADPH oxidase. *Life Sciences*.

[10] Christ M, Bauersachs J, Liebetrau C, Heck M, Günther A, Wehling M (2002). Glucose increases endothelial-dependent superoxide formation in coronary arteries by NAD(P)H oxidase activation. *Diabetes*.

[11] Inoue I, Goto S-I, Mizotani K (2000). Lipophilic HMG-CoA reductase inhibitor has an anti-inflammatory effect: reduction of mRNA levels for interleukin-1*β*, interleukin-6, cyclooxygenase-2, and p22phox by regulation of peroxisome proliferator-activated receptor *α* (PPAR*α*) in primary endothelial cells. *Life Sciences*.

[12] Schoonjans K, Staels B, Auwerx J (1996). The peroxisome proliferator activated receptors (PPARs) and their effects on lipid metabolism and adipocyte differentiation. *Biochimica et Biophysica Acta*.

[13] Willson TM, Brown PJ, Sternbach DD, Henke BR (2000). The PPARs: from orphan receptors to drug discovery. *Journal of Medicinal Chemistry*.

[14] Gearing KL, Gottlicher M, Teboul M, Widmark E, Gustafsson JA (1993). Interaction of the peroxisome-proliferator-activated receptor and retinoid X receptor. *Proceedings of the National Academy of Sciences of the United States of America*.

[15] Mandard S, Müller M, Kersten S (2004). Peroxisome proliferator-activated receptor *α* target genes. *Cellular and Molecular Life Sciences*.

[16] Kersten S (2008). Peroxisome proliferator activated receptors and lipoprotein metabolism. *PPAR Research*.

[17] Aoki T, Yoshinaka Y, Yamazaki H (2002). Triglyceride-lowering effect of pitvastatin in a rat model of postprandial lipemia. *European Journal of Pharmacology*.

[18] Nicholls SJ, Tuzcu EM, Sipahi I (2006). Effects of obesity on lipid-lowering, anti-inflammatory, and
antiatherosclerotic benefits of atorvastatin or pravastatin in
patients with coronary artery disease (from the REVERSAL Study). *The American Journal of Cardiology*.

[19] Kostapanos MS, Milionis HJ, Lagos KG, Rizos CB, Tselepis AD, Elisaf MS (2008). Baseline triglyceride levels and insulin sensitivity are major determinants of the increase of LDL particle size and buoyancy induced by rosuvastatin treatment in patients with primary hyperlipidemia. *European Journal of Pharmacology*.

[20] Planavila A, Laguna JC, Vazquez-Carrera M (2005). Atorvastatin improves peroxisome proliferator-activated receptor signaling in cardiac hypertrophy by preventing nuclear factor-*κ*B activation. *Biochimica et Biophysica Acta*.

[21] Zapolska-Downar D, Siennicka A, Kaczmarczyk M, Kołodziej B, Naruszewicz M (2004). Simvastatin modulates TNF*α*-induced adhesion molecules expression in human endothelial cells. *Life Sciences*.

[22] Landrier J-F, Thomas C, Grober J (2004). Statin induction of liver fatty acid-binding protein (L-FABP) gene expression is peroxisome proliferator-activated receptor-*α*-dependent. *The Journal of Biological Chemistry*.

[23] Torra IP, Jamshidi Y, Flavell DM, Fruchart J-C, Staels B (2002). Characterization of the human PPAR*α* promoter: identification of a functional nuclear receptor response element. *Molecular Endocrinology*.

[24] Inoue I, Hayashi K, Yagasaki F (2003). Apoptosis of endothelial cells may be mediated by genes of peroxisome proliferator-activated receptor *γ*1 (PPAR*γ*1) and PPAR*α* genes. *Journal of Atherosclerosis and Thrombosis*.

[25] Inoue I, Shinoda Y, Ikeda M (2005). CLOCK/BMAL1 is involved in lipid metabolism via transactivation of the peroxisome proliferator-activated receptor (PPAR) response element. *Journal of Atherosclerosis and Thrombosis*.

[26] Lundbye JB, Thompson PD (2005). Statin use in the metabolic syndrome. *Current Atherosclerosis Reports*.

[27] Palinski W (2001). New evidence for beneficial effects of statins unrelated to lipid lowering. *Arteriosclerosis, Thrombosis, and Vascular Biology*.

[28] Kleemann R, Princen HMG, Emeis JJ (2003). Rosuvastatin reduces atherosclerosis development beyond and independent of its plasma cholesterol-lowering effect in APOE*3-Leiden transgenic mice: evidence for antiinflammatory effects of rosuvastatin. *Circulation*.

[29] Grip O, Janciauskiene S, Lindgren S (2002). Atorvastatin activates PPAR-*γ* and attenuates the inflammatory response in human monocytes. *Inflammation Research*.

[30] Gottlicher M, Widmark E, Li Q, Gustafsson JA (1992). Fatty acids activate a chimera of the clofibric acid-activated receptor and the glucocorticoid receptor. *Proceedings of the National Academy of Sciences of the United States of America*.

[31] Lake BG (1995). Mechanisms of hepatocarcinogenicity of peroxisome-proliferating drugs and chemicals. *Annual Review of Pharmacology and Toxicology*.

[32] Dowell P, Ishmael JE, Avram D, Peterson VJ, Nevrivy DJ, Leid M (1997). p300 functions as a coactivator for the peroxisome proliferator-activated receptor *α*. *The Journal of Biological Chemistry*.

[33] Zhu Y, Qi C, Jain S, Rao MS, Reddy JK (1997). Isolation and characterization of PBP, a protein that interacts with peroxisome proliferator-activated receptor. *The Journal of Biological Chemistry*.

[34] Puigserver P, Wu Z, Park CW, Graves R, Wright M, Spiegelman BM (1998). A cold-inducible coactivator of nuclear receptors linked to adaptive thermogenesis. *Cell*.

[35] Glass CK (2006). Going nuclear in metabolic and cardiovascular disease. *Journal of Clinical Investigation*.

